# De novo biosynthesis of α‐aminoadipate via multi‐strategy metabolic engineering in *Escherichia coli*

**DOI:** 10.1002/mbo3.1301

**Published:** 2022-09-01

**Authors:** Yang Zhang, Meng Liu, Bingqi Cai, Keqin He, Meng Wang, Biqiang Chen, Tianwei Tan

**Affiliations:** ^1^ Beijing Key Laboratory of Bioprocess, National Energy R&D Center for Biorefinery Beijing University of Chemical Technology Beijing People's Republic of China

**Keywords:** *Escherichia coli*, metabolic engineering, multi‐strategy regulation, α‐aminoadipate

## Abstract

As a non‐protein amino acid, α‐aminoadipate is used in the fields of medicine, chemical engineering, food science, and others. For example, α‐aminoadipate is an important precursor for the production of β‐lactam antibiotics. Currently, the synthesis of α‐aminoadipate depends on chemical catalysis that has the disadvantages of high cost, low yield, and serious pollution. In this study, we construct a biosynthesis pathway of α‐aminoadipate in *Escherichia coli* using lysine as a precursor. In addition, we regulate the cell metabolism to improve the titer of α‐aminoadipate via multi‐strategy metabolic engineering. First, a novel synthetic pathway was constructed to realize de novo synthesis of α‐aminoadipate with titers of 82 mg/L. Second, the key enzymes involved in enhancing precursor synthesis were overexpressed and the CO_2_ fixation process was introduced, and these led to 80% and 34% increases in the α‐aminoadipate concentration, reaching 147 and 110 mg/L, respectively. Third, cofactor regulation was used to maintain the coupling balance of material and energy, with the intracellular α‐aminoadipate concentration reaching 140 mg/L. Fourth, the weakening of the synthesis of acetic acid was used to strengthen the synthesis of α‐aminoadipate, and this resulted in the enhancement of the α‐aminoadipate concentration by 2.2 times, reaching 263 mg/L. Finally, combination optimization was used to promote the production of α‐aminoadipate. The titers of α‐aminoadipate reached 368 mg/L (strain EcN11#) and 415 mg/L (strain EcN11##), which was 3.5 and 4 times higher than that of the parent strain. With these efforts, 1.54 g/L of α‐aminoadipate was produced under fed‐batch conditions by strain EcN11#. This study is the first to present the effective biosynthesis of α‐aminoadipate in *E. coli* using multi‐strategy metabolic engineering.

## Graphical abstract

A biosynthetic pathway was constructed to produce α‐aminoadipate. Multi‐strategy metabolic engineering was used for regulating cell metabolism. Finally, 1.54 g/L of α‐aminoadipate was produced in fed‐batch experiments.

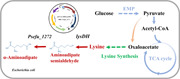

## 1 INTRODUCTION

Note, α‐aminoadipate is a non‐protein amino acid that was first discovered in corn seeds and the urine of humans or guinea pigs. And, α‐aminoadipate is the precursor for the synthesis of lysine in fungi. However, in plants and mammals, α‐aminoadipate is produced by lysine degradation (K. Zhang et al., 2010). Also α‐aminoadipate has several applications. For example, it is used for the treatment of eye diseases because it can specifically act on retinal Mullerian cells. It is also used in the chemical synthesis of methotrexate derivatives that can be effectively used as anti‐rheumatic and anticancer agents. Plus, α‐aminoadipate is also a precursor in the fermentation production of β‐lactam antibiotics (such as penicillin and cephalosporin) (Weber et al., 2012). In addition, α‐aminoadipate is a specific toxin of astrocytes and an important compound for studying the role of astrocytes in growth and disease (Brown & Kretzschma, 1998). Currently, α‐aminoadipate is still synthesized using chemical methods that produce low yields, cause serious environmental pollution, and are not cost‐effective. Therefore, we hope to realize the green synthesis of α‐aminoadipate by constructing microbial cell factories.

The design and construction of new cascade reactions in microorganisms for biosynthesis have been proven to be feasible in many studies, and this process can supply cofactors for product synthesis (M. Wang, Chen, et al., 2017) and serve as microbial cell factories to produce high value‐added chemicals (Ko et al., 2020). As a C_6_ amino acid, α‐aminoadipate has a structure similar to that of lysine. We hope to use lysine as a precursor to finding a suitable enzyme to convert lysine to α‐aminoadipate. Therefore, we will summarize the biosynthesis and degradation pathways of lysine. In microorganisms, the synthesis of lysine has two completely different pathways: one is the diaminopimelic acid (DAP) pathway, and the other is the α‐aminoadipate pathway. The DAP pathway exists in bacteria, fungi, and plants, and it uses aspartate as a precursor to synthesize lysine through a 10 step enzymatic reaction, including aspartate transaminase (encoded by *aspC*), aspartate kinase (encoded by *lysC*), aspartate semialdehyde dehydrogenase (encoded by *asd*), 4‐hydroxy‐tetrahydrodipicolinate synthase (encoded by *dapA*), 4‐hydroxy‐tetrahydrodipicolinate synthase (encoded by *dapB*), 2,3,4,5‐tetrahydropyridine‐2,6‐dicarboxylate *N*‐succinyltransferase (encoded by *dapD*), *N*‐succinyldiaminopimelate aminotransferase (encoded by *dapC*), succinyl‐diaminopimelate desuccinylase (encoded by *dapE*), diaminopimelate epimerase (encoded by *dapF*), and diaminopimelate decarboxylase (encoded by *lysA*) (H. Xu et al., 2006). The DAP biosynthetic pathway has been extensively studied in *Escherichia coli* and *Corynebacterium glutamicum* and has been engineered for the industrial production of lysine. Unlike the DAP pathway in *E. coli*, there is a multifunctional enzyme in *C. glutamicum* (meso‐diaminopimelate dehydrogenase, encoded by the gene *ddh*) that can replace *DapD*, *DapC*, *DapE*, and *DapF* to directly reduce l‐piperidine‐2,6‐dicarboxylic acid to D, l‐diaminopimelate (Ishino et al., 1984; and Misono et al., 1986). The α‐aminoadipate pathway exists in eucalyptus and higher fungi that use ketoglutarate as the precursor, and it is gradually catalyzed by homoisocitrate synthase, cis‐aconitase, and isocitrate dehydrogenase to produce α‐aminoadipate. Next, α‐aminoadipate is catalyzed by α‐aminoadipate reductase, saccharopine dehydrogenase, and saccharopine reductase to generate lysine (Burk et al., 2007; Hermann, 2003; Zabriskie & Jackson, 2000). However, lysine is degraded by the reverse α‐aminoadipate pathway in plants and animals (Arruda et al., 1982, 2000). It has been reported that lysine is catalytically converted to saccharopine by lysine‐ketoglutarate reductase (LKR) and then catalytically converted to α‐aminoadipate semialdehyde by saccharopine dehydrogenase (SDH). Finally, α‐aminoadipate semialdehyde is catalytically converted to α‐aminoadipate by α‐aminoadipate semialdehyde dehydrogenase (Arruda et al., 2000; de Mello Serrano et al., 2012). These above enzymes probably originated from *Proteusbacillus vulgaris* and are also found in animals and plants. It has been determined that lysine is directly converted to α‐aminoadipate semialdehyde by lysine dehydrogenase with NAD(P)+ as a cofactor in prokaryotes. It is then oxidized to α‐aminoadipate (Misono & Nagasaki, 1982).

**Figure 1:**
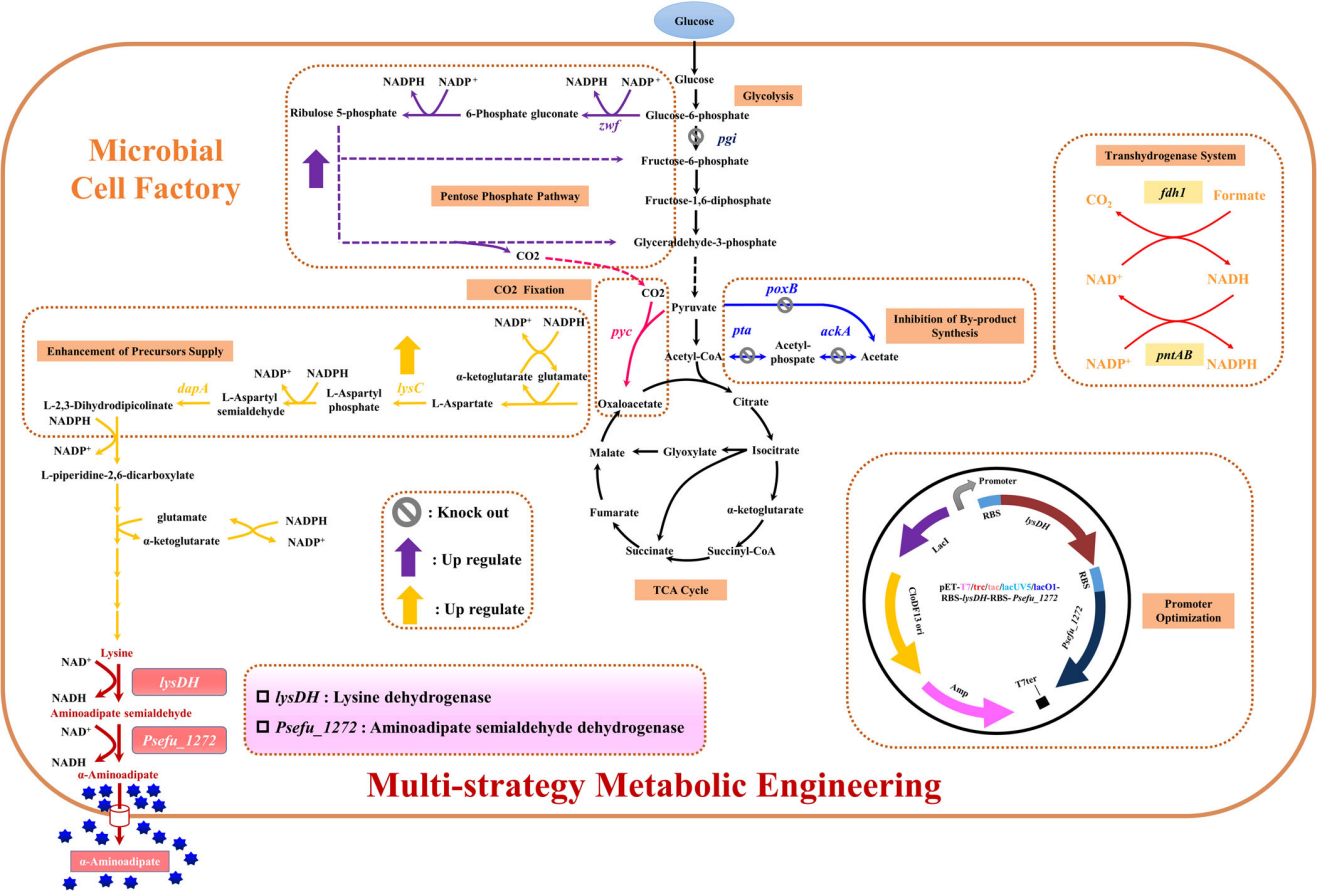
Scheme of the multi‐strategy metabolic engineering of the α‐aminoadipate biosynthesis system. *ackA*, encoding acetate kinase; *dapA*, encoding 4‐hydroxy‐tetrahydrodipicolinate synthase; *fdh1*, encoding formate dehydrogenase; *lysC*, encoding aspartate kinase; nadK, encoding NAD kinase; *pgi*: encoding glucose‐6‐phosphate isomerase; *poxB*, encoding pyruvate oxidase; *pta*, encoding phosphate acetyltransferase; *pyc*, encoding pyruvate carboxylase; *pntAB*, encoding NADP transhydrogenase; *zwf*, encoding glucose‐6‐phosphate 1‐dehydrogenase.

In summary, α‐aminoadipate is both the precursor for lysine synthesis and an intermediate for lysine degradation. Therefore, in this study, we combine the DAP pathway with the reverse α‐aminoadipate pathway to design a biosynthesis pathway for α‐aminoadipate (Figure 1). In this study, we select *E. coli* as the host and glucose as the carbon source to produce α‐aminoadipate by fermentation through the above pathway. And, multi‐strategy metabolic engineering was used to regulate microbial cell factories to realize the efficient and green synthesis of α‐aminoadipate.

## 2 MATERIALS AND METHODS

### 2.1 Flux balance analysis (FBA) and OptForce on iML1515

Both FBA (Orth et al., 2010) and OptForce algorithm (Ranganathan et al., 2010) were performed using the Gurobi and COBRApy toolkit via Python to determine the flux spans of each reaction. The glucose uptake rate was set at 10 mmol/gDCW/h. The upper limit for biomass in the overproducing strain was set to 5% of the theoretical maximum for biomass flux.

### 2.2 Media, strains, plasmids, materials, and growth conditions

The *E. coli* Trans 10 and its derivatives were cultured in the lysogeny broth (LB) medium with 200 rpm at 37°C, which contained 5 g yeast extract, 5 g NaCl, and 10 g tryptone per liter. The recombinant *E. coli* strains were cultured in the M9 medium with 200 rpm at 37°C for feeding experiments or de novo production for culturing cells and at 30°C for inducing gene expression. The M9 medium contained 10 g glucose, 3 g yeast extract, 1 g NH4Cl, 6.78 g Na_2_HPO_4_, 3 g KH_2_PO_4_, and 0.5 g NaCl per liter. For the fed‐batch cultivation, culture conditions referred to the strategy described in Li et al. (2019). Ampicillin, kanamycin, chloramphenicol, or spectinomycin was added to screen the recombinant strains with final concentrations of 100, 50, 30, or 50 mg/L, respectively.

The *E. coli* JM109(DE3) was used as the host strain to produce α‐aminoadipate. The kits for genomic DNA isolation, plasmid extraction, and DNA recovery were purchased from Omega Bio‐Tek, and all of the enzymes were purchased from New England Biolabs. All of the strains and plasmids used are listed in Table A1. The knockout strains were constructed using RED recombination.

### 2.3 Plasmid construction

All of the primers used for gene cloning are listed in Table A2. The l‐lysine 6‐dehydrogenase encoding gene *lysDH* from *Bacillus thermoamylovorans 1A1* (GenBank: CEE01557.1) and the aminoadipate semialdehyde dehydrogenase coding gene *Psefu_1272* (GenBank: AEF21248.1) from *Pseudomonas fulva 12‐X* were codon‐optimized for *E. coli*, which were synthesized and respectively ligated into the plasmids pUC57‐*lysDH* and pUC57‐*pse* by BGI. Both *lysDH* and *Psefu_1272* were amplified by polymerase chain reaction (PCR) using pUC57‐*lysDH* and pUC57‐*pse* as the templates, respectively. The PCR products were subcloned into plasmid pETDuet‐1 to generate the plasmid pET‐T7‐*lysDH‐pse* by the Gibson assembly reaction (Gibson et al., 2009). Then, the plasmids pET‐tac‐*lysDH‐pse*, pET‐trc‐*lysDH‐pse*, pET‐lacUV5‐*lysDH‐pse*, and pET‐LacO1‐*lysDH‐pse* were obtained by replacing the T7 promoter with the different promoters.

### 2.4 Measurement of the biomass, substrate consumption rate, and metabolic products

The cell density was determined by measuring the turbidity of the culture medium at 600 nm using a spectrophotometer (Thermo Fisher Scientific).

The concentrations of glucose and organic acids were determined by the UltiMate 3000 HPLC (Thermo Fisher Scientific) using the following apparatus and operating conditions: Bio‐Rad Aminex HPX‐87H column (Bio‐Rad Laboratories) with RID and UV detectors; column temperature of 65°C; and 0.6 mL min^−1^ of 5 mM sulfuric acid as the mobile phase. The concentration of α‐aminoadipate was analyzed by LC‐MS using the AB SCIEX QTRAP 5500 system (AB SCIEX) equipped with a HILIC‐Z column (2.1 × 100 mm) (Agilent Technologies). The mobile phase was: A: water containing 0.1% (v/v) formic acid, B: acetonitrile containing 0.1% (v/v) formic acid, and 20 mM ammonium acetate with a flow velocity of 0.3 mL min^−1^ at 40°C. Also, α‐aminoadipate was further identified by gas chromatography‐mass spectrometry (GC‐MS) using the Agilent Technologies 7890B‐5977A system (Agilent Technologies) in which the appropriate amount of fermentation supernatant was dried by vacuum centrifugation or freeze‐drying and extracted using a silylation reagent at a ratio of *N*, *O*‐bis‐(trimethylsilyl) trifluoroacetamide to trimethylchlorosilane of 99:1.

## 3 RESULTS AND DISCUSSION

### 3.1 Designing an optimal *E. coli* α‐aminoadipate producer via FBA and OptForce

**Figure 2:**
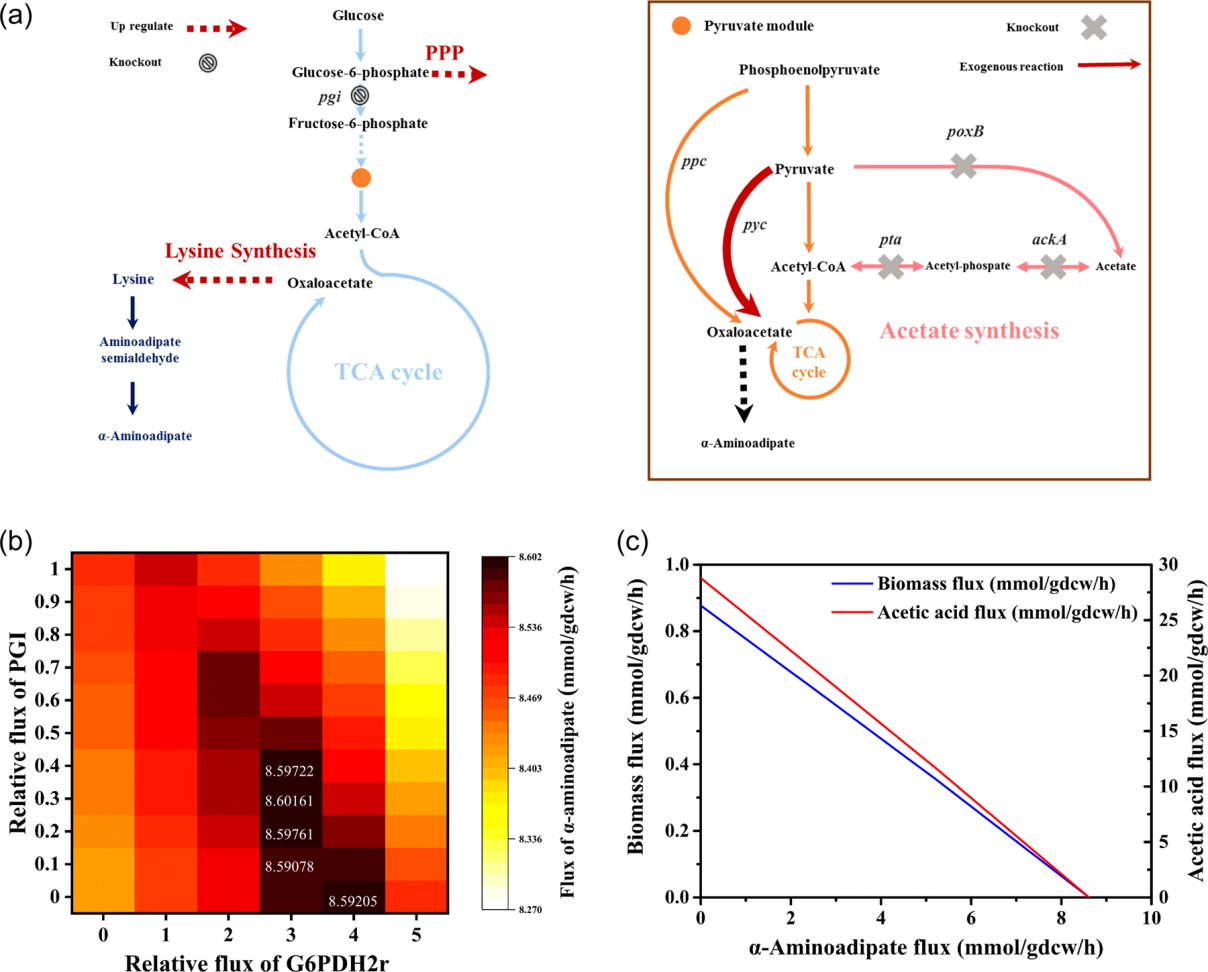
α‐Aminoadipate overproducing strategies in recombinant *Escherichia coli*. (a) Metabolic interventions prediction via OptForce based on iML1515. *ackA*, encoding acetate kinase; *poxB*, encoding pyruvate oxidase; *pgi*, encoding glucose‐6‐phosphate isomerase; *ppc*, encoding phosphoenolpyruvate carboxylase; *pta*, encoding phosphate acetyltransferase; *pyc*, encoding pyruvate carboxylase. (b) The influences of glycolysis and the PPP flux on the α‐aminoadipate flux. G6PDH2r, the reaction catalyzed by glucose‐6‐phosphate 1‐dehydrogenase (encoded by *zwf*); PGI, the reaction catalyzed by glucose‐6‐phosphate isomerase (encoded by *pgi*). (c) The effect of the biomass flux and the acetate flux on α‐aminoadipate flux; gdcw, gram of dry cell weight.

We added two reactions catalyzed by lysine dehydrogenase and aminoadipate semialdehyde dehydrogenase and the α‐aminoadipate exchange reaction to iML1515. Both FBA and OptForce were used to calculate and predict the metabolic strategies of α‐aminoadipate overproduction. As shown in Figure 2a, nine reactions in the lysine biosynthesis pathway were found to require amplification. The pentose phosphate pathway (PPP) was required to be upregulated because the PPP could provide NADPH for lysine synthesis. In addition, we introduced the exogenous reaction catalyzed by pyruvate carboxylase (encoded by pyc) into the model and calculated the theoretical yield of α‐aminoadipate. When the above reaction was added to the model, the yield was 90% (mol/mol), which was 9.8% higher than that of the wild type (the yield was 82% [mol/mol]). This is because pyruvate carboxylase enhanced the carbon flux of α‐aminoadipate biosynthesis by the CO_2_ fixation. The reaction of glucose 6‐phosphate to fructose 6‐phosphate catalyzed by glucose‐6‐phosphate isomerase (encoded by *pgi*) in glycolysis is the competitive pathway of PPP, which is required to be downregulated. The α‐aminoadipate flux was calculated at different levels of the PPP (in terms of G6PDH2r) and the glycolysis pathway (in terms of PGI). In Figure 2b, the higher α‐aminoadipate flux was obtained when the PGI flux was decreased and the PPP was upregulated. In addition, Figure 2c shows the negative effect of the biomass and the acetate pathway on α‐aminoadipate flux.

### 3.2 Constructing the bioproduction pathway of α‐aminoadipate in *E. coli*

Lysine dehydrogenase with NAD(P)^+^ as a cofactor in prokaryotes can directly convert lysine into aminoadipate semialdehyde (Misono & Nagasaki, 1982). Subsequently, aminoadipate semialdehyde is converted to α‐aminoadipate by aminoadipate semialdehyde dehydrogenase. Searching through the UniProt database (https://www.uniprot.org/), we selected lysine dehydrogenase encoded by the *lysDH* gene from *Bacillus thermoamylovorans 1A1* and aminoadipate semialdehyde dehydrogenase encoded by the *Psefu_1272* gene from *Pseudomonas fulva 12‐X* to construct the biosynthetic pathway of α‐aminoadipate in *E. coli* (Figure 1).

**Figure 3:**
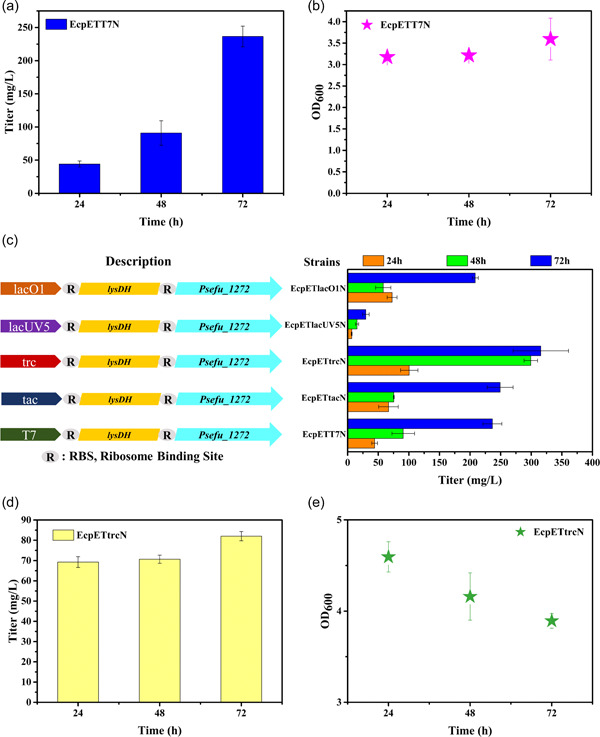
Optimization of α‐aminoadipate production. (a) The titers of α‐aminoadipate from lysine by the feeding experiment. (b) and (e) The biomass at different times. (c) The titers of α‐aminoadipate from lysine at different promoters. (d) The de novo biosynthesis of α‐aminoadipate by the strain EcpETtrcN from glucose.

**Figure B1:**
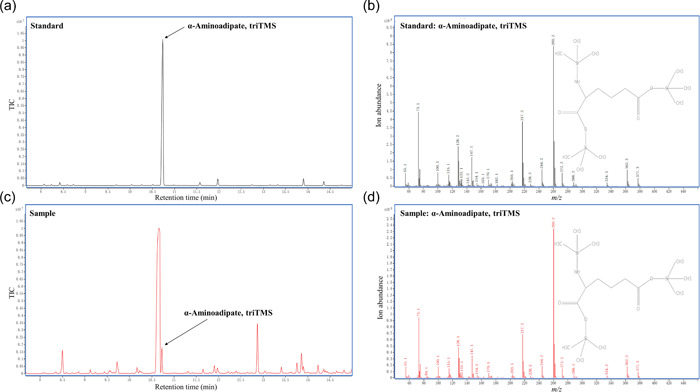
Identification of α‐aminoadipate by gas chromatography‐mass spectrometry (GC‐MS). (a) The chromatogram for the standard of α‐aminoadipate, triTMS. (b) The mass spectrum for the standard of α‐aminoadipate, triTMS. (c) The chromatogram for the sample of α‐aminoadipate, triTMS. (d) The mass spectrum for the sample of α‐aminoadipate, triTMS. TIC, total ion chromatogram.

The *lysDH* from *Bacillus thermoamylovorans 1A1* and *Psefu_1272* from *Pseudomonas fulva 12‐X* were codon‐optimized for *E. coli* and ligated into the pETDuet‐1 plasmid. Their expressions were induced using isopropyl β‐d‐1‐thiogalactopyranoside (IPTG). The *E. coli* JM109(DE3) cells harboring the pET‐T7‐*lysDH‐pse* plasmid were designated as strain EcpETT7N, and this strain produced 236.5 mg/L of α‐aminoadipate in M9 at 72 h by feeding experiments (feeding lysine at a final concentration of 5 g L^−1^ to the M9 medium) (Figure 3a). The product was verified by GC‐MS, and the result showed that the product from the fermentation broth was α‐aminoadipate (Figure B1).

To determine the optimal expression level of heterologous enzymes catalyzing the reactions for α‐aminoadipate synthesis in *E. coli*, the *lysDH*, and *Psefu_1272* genes were ligated into plasmids with different promoters that included the tac, trc, lac UV5, and lacO1 promoters. Thus, α‐aminoadipate titers produced by feeding experiments with the recombinant strains of EcpETtacN, EcpETtrcN, EcpETlacUV5N, and EcpETlacO1N (Table A1) were compared. The results showed that the trc promoter (pET‐trc‐*lysDH‐pse*) was more suitable for α‐aminoadipate production than the other promoters. The strain EcpETtrcN effectively converted lysine to α‐aminoadipate with titers of 315.5 mg/L (Figure 3c). To explore the de novo biosynthesis of α‐aminoadipate, the strain EcpETtrcN was cultivated in an M9 medium with 10 g L^−1^ of glucose, which produced 82 mg/L of α‐aminoadipate at 72 h (Figure 3d).

### 3.3 Increasing the supply of precursors and introducing the CO_2_ fixation process

According to the results from FBA and OptForce, the lysine synthesis pathway should be upregulated. In *E. coli*, lysine is synthesized through the DAP pathway that involves two key enzymes, namely, aspartate kinase (encoded by *lysC*) and 4‐hydroxy‐tetrahydrodipicolinate synthase (encoded by *dapA*) (Contador et al., 2009; J. Wang et al., 2015; J. Xu et al., 2014). Some studies have shown that aspartate kinase is inhibited by the feedback of lysine. We overexpressed the above two genes to strengthen the two key enzymes. In addition, the meso‐diaminopimelate dehydrogenase from *Corynebacterium glutamicum*can replace the functions of four enzymes (2,3,4,5‐tetrahydropyridine‐2,6‐dicarboxylate *N*‐succinyltransferase, *N*‐succinyldiaminopimelate aminotransferase, succinyl‐diaminopimelate desuccinylase, diaminopimelate epimerase) in *E. coli* to directly convert l‐piperidine‐2,6‐dicarboxylic acid into d, l‐diaminopimelate. Therefore, we hoped to replace the original four‐step enzyme by introducing meso‐diaminopimelate dehydrogenase (encoded by *ddh*) from *Corynebacterium glutamicum* ATCC 13032 to shorten the metabolic pathway.

**Figure 4:**
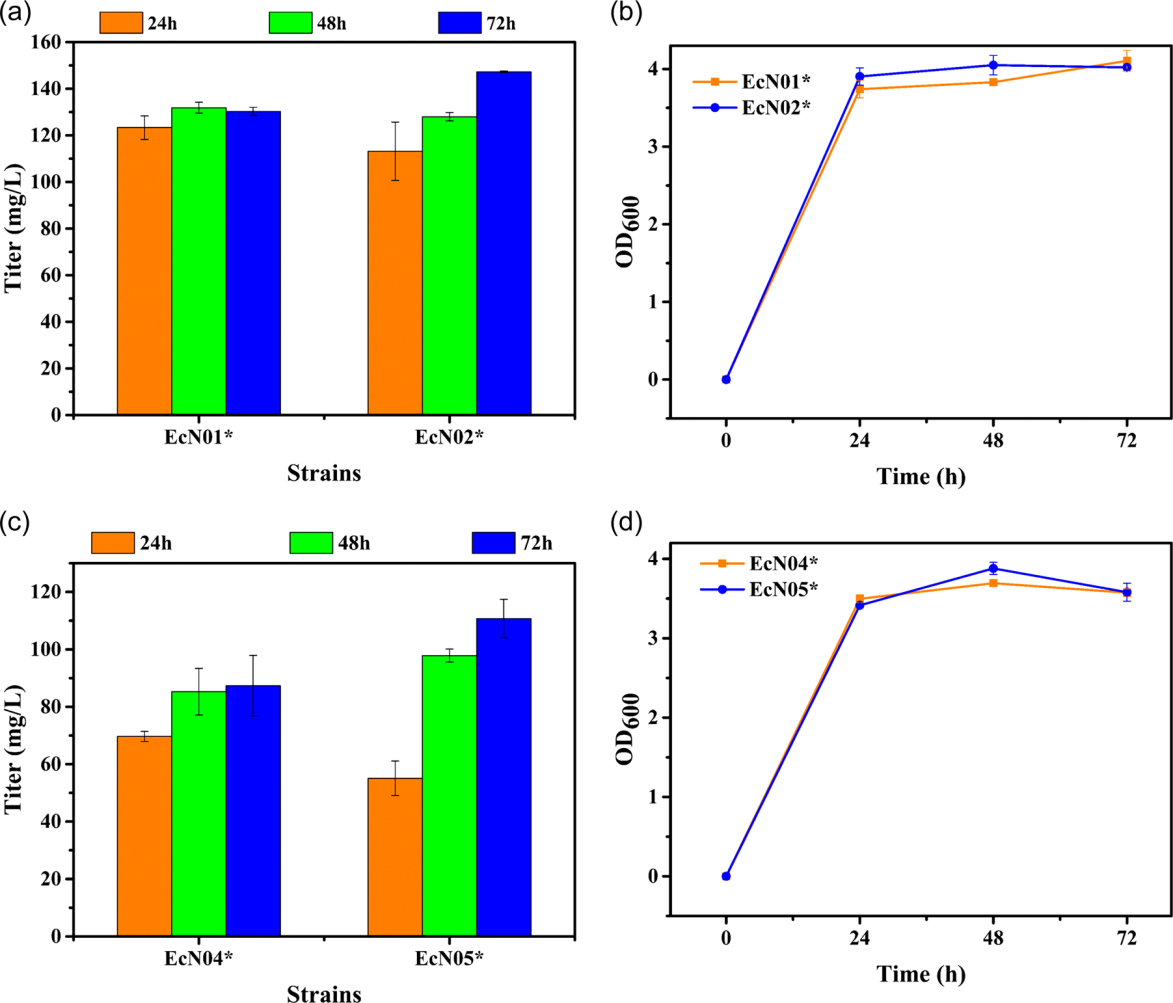
Enhancement of the precursor supply and introduction of the CO_2_ fixation process for promoting α‐aminoadipate production. (a) and (c) The titers of α‐aminoadipate in the different strains. (b) and (d) The biomass in the different strains.

**Figure B2:**
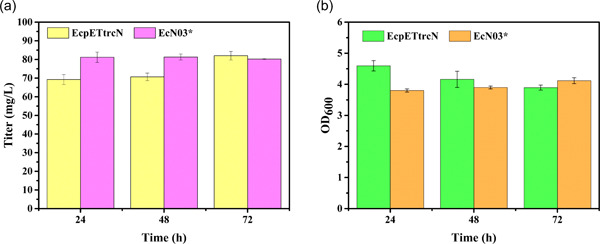
Overexpression of meso‐diaminopimelate dehydrogenase (encoded by *ddh*) from *Corynebacterium glutamicum* ATCC 13032 (a) The titers of α‐aminoadipate. (b) The biomass in the different strains.

Five candidate genes derived from *E. coli* or *Corynebacterium glutamicum* were constructed in the plasmid pACYC‐tac‐T7‐T7ter or pACYCDuet‐1 and electrotransformed into the strain EcpETtrcN to obtain the strain EcN01*‐EcN03* (Table A1). The results of fermentation in the shake flasks showed that the titer of α‐aminoadipate produced by the strain EcN02* was higher, reaching 147 mg/L (Figure 4a). These results demonstrated that overexpression of *lysC* and *dapA* promoted the synthesis of α‐aminoadipate. The mutant *lysCT311I* with anti‐feedback inhibition and *dapA* from *Corynebacterium glutamicum* ATCC 13032 were better than the *lysC* and *dapA* from *E. coli*, and the titer of α‐aminoadipate was 80% higher than that of the strain EcpETT7N. Next, we hoped to introduce meso‐diaminopimelate dehydrogenase (encoded by *ddh*) from *Corynebacterium glutamicum* ATCC 13032 to shorten the synthesis pathway of the precursor lysine. However, the strain EcN03* produced the same amount of α‐aminoadipate compared with the strain EcpETtrcN (Figure B2), speculating that the insufficient supply of cofactors and the prior balance may have been disrupted.

**Figure B3:**
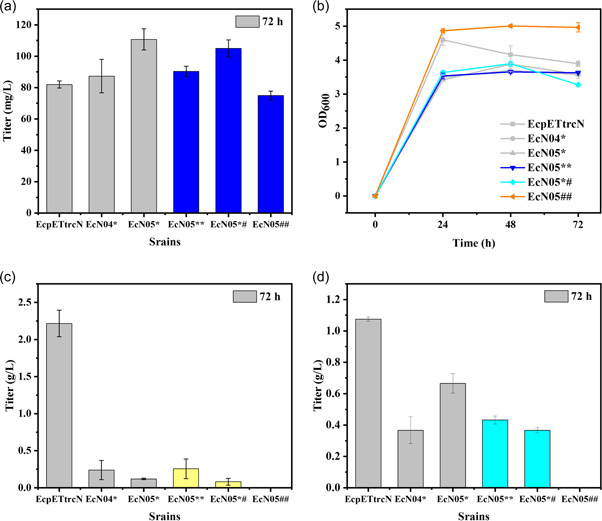
Comparison of the effects of overexpression of *ppc* and expression of *pyc* on the synthesis of α‐aminoadipate. (a) The titers of α‐aminoadipate in the different strains. (b) The biomass in the different strains. (c) The titers of acetic acid. (d) The titers of glucose.

In addition, it has been confirmed that oxaloacetate is another important precursor for the synthesis of lysine or lysine derivatives. Increasing the replenishment of oxaloacetate is beneficial for improving lysine and lysine derivative production. Pyruvate carboxylase catalyzes the transformation of pyruvate to oxaloacetate, and the overexpression of its coding gene, *pyc*, can enhance the supply of oxaloacetate. The mutant *pycP458S* is a beneficial mutation that can improve the activity of pyruvate carboxylase (Xiong et al., 2021). And FBA calculation results also showed that the theoretical yield of α‐aminoadipate increased by nearly 10% after the introduction of pyruvate carboxylase. In addition, the catalytic reaction by pyruvate carboxylase is a CO_2_ fixation process, which could achieve the intracellular CO_2_ reutilization to enhance the carbon flux of α‐aminoadipate biosynthesis. Thus, *pyc* from *Corynebacterium glutamicum* ATCC 13032 and its mutant *pycP458S* were constructed in the plasmid pRSF‐tac‐T7‐T7ter to obtain plasmids pRSF‐tac‐*pyc* and pRSF‐tac‐*pycP458S* and to finally obtain the recombinant strains EcN04* and EcN05*. The results of the shake flask fermentation showed that expression of the *pyc* gene mutant (*pycP458S*) encoding pyruvate carboxylase from *Corynebacterium glutamicum* ATCC 13032 could promote the synthesis of α‐aminoadipate. The titer of α‐aminoadipate reached 110 mg/L (Figure 4c), which was 34% higher than that of the strain EcpETtrcN. Furthermore, endogenous phosphoenolpyruvate carboxylase encoded by *ppc* catalyzed the conversion of phosphoenolpyruvate (PEP) to oxaloacetate. However, the glucose transport system of *E. coli* was mainly the phosphoenolpyruvate‐carbohydrate phosphotransferase system (PTS), which consumed PEP to assist glucose transport and produce pyruvate. Therefore, to increase the availability of oxaloacetate, expressing *pyc* instead of *ppc* was probably better. Some experiments were carried out to compare the effects of overexpression of *ppc* and expression of *pyc* on the synthesis of α‐aminoadipate. The results of the shake flask fermentation showed that overexpression of *ppc* also could promote the synthesis of α‐aminoadipate. Among them, the titers of α‐aminoadipate of the strain EcN05*# are equivalent to that of the strain EcN05*. The biomass and glucose utilization were also similar. However, coexpression of *ppc* and *pyc* was detrimental to the synthesis of α‐aminoadipate (strain EcN05##). The coexpression of *ppc* and *pyc* resulted in the imbalance of upstream and downstream metabolic flux. Although it promoted the utilization of glucose, excessive oxaloacetate did not effectively enter the synthesis pathway of α‐aminoadipate, but promoted the TCA cycle, increasing the biomass of strain EcN05## (Figure B3).

### 3.4 Regulating intracellular cofactors

Cofactors (eg., NADH, NADPH, and ATP) are important metabolic factors in microbial cells. As the most important redox carriers in cell metabolism, NADH/NAD^+^ and NADPH/NADP^+^ not only act as electron acceptors for catalyzing substrate catabolism but also provide reducing power for energy‐dependent redox reactions. ATP/ADP from substrate‐level phosphorylation and oxidative phosphorylation can enter the metabolic network of microorganisms in a variety of ways, such as substrates, products, activators, or inhibitors, to control the physiological function of cells and participate in the formation of the cytoskeleton system. Therefore, the control of the intracellular cofactor balance is a basic requirement to maintain the normal metabolism of cells and achieve a balance of material and energy couple. Currently, the primary regulation strategies of cofactors can be divided into four categories: regulation of endogenous cofactor systems, regulation of heterologous cofactor regeneration systems, modification of cofactor preferences, and creation of synthetic‐cofactor systems (M. Wang, Chen, et al., 2017; Y. Zhang et al., 2021).

**Figure 5:**
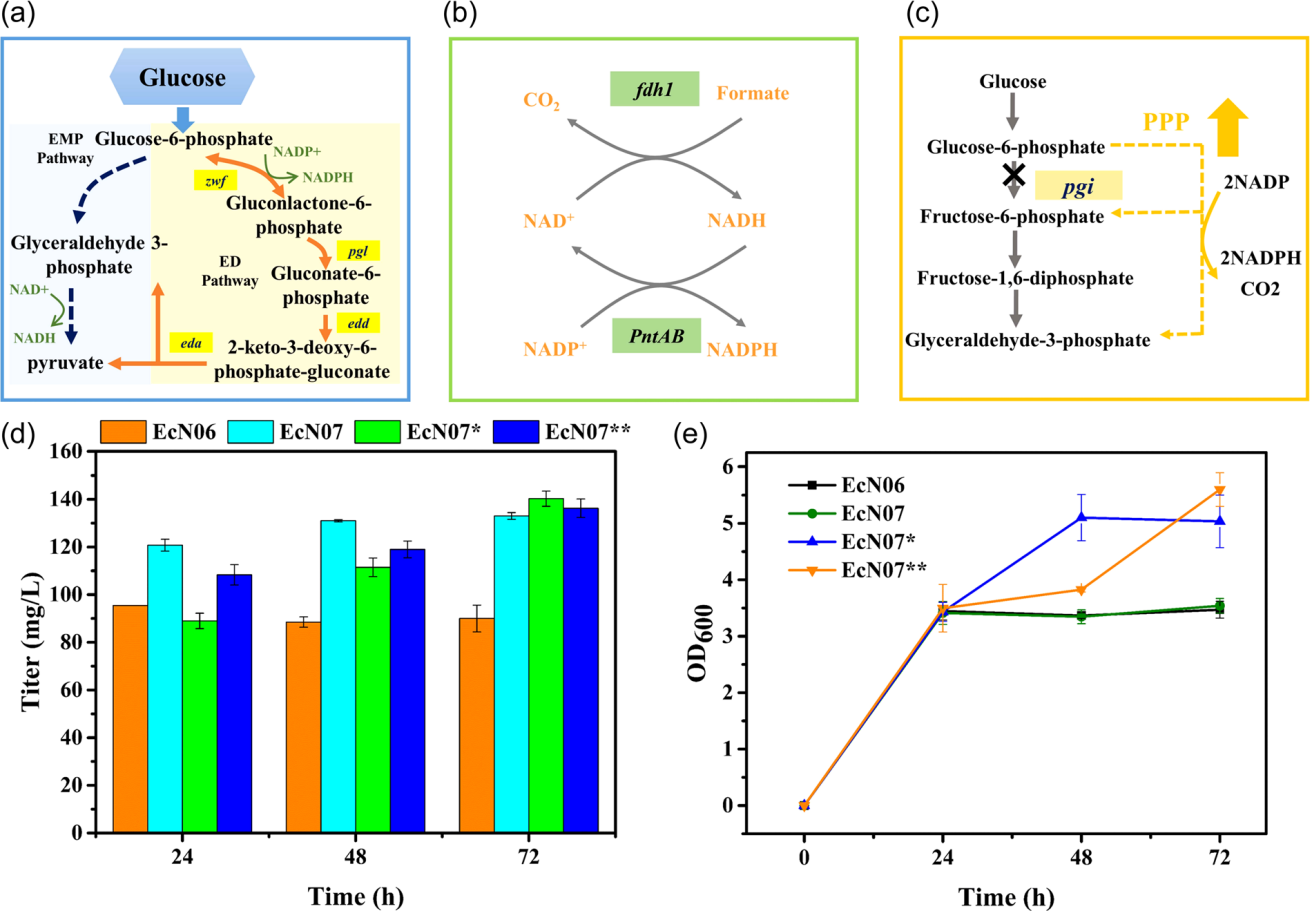
The effect of different cofactor regulation strategies on α‐aminoadipate synthesis. (a) The Entner–Doudoroffed pathway. (b) The transhydrogenase system. (c) The knockout of the *pgi* gene (encoding glucose‐6‐phosphate isomerase) for enhancing the pentose phosphate pathway. (d) The titers of α‐aminoadipate in different strains. (e) The biomass in the different strains.

In the synthesis pathway of α‐aminoadipate constructed in this study, the synthesis of the precursor lysine required a significant amount of NADPH, and the conversion of lysine to aminoadipic acid required NAD^+^ as the cofactor. Therefore, it was necessary to regulate the level of intracellular cofactors to achieve cofactor balance to promote the synthesis of α‐aminoadipate. In this study, the cofactor level was regulated by three strategies: (1) introducing the Entner–Doudoroffed pathway (ED pathway, Figure 5a) (M. Wang, Zhou, et al., 2017); (2) introducing the transhydrogenase system (Figure 5b) (Y. Chen et al., 2015); and (3) enhancing the pentose phosphate pathway (by deleting the *pgi* gene that encodes glucose‐6‐phosphate isomerase, Figure 5c) (Marx et al., 2003). The recombinant strains EcN06, EcN07, EcN07*, and EcN07** were obtained. The fermentation results showed that the introduction of the transhydrogenase system and the strengthening of the pentose phosphate pathway promoted the synthesis of α‐aminoadipate. Then, the strains EcN07, EcN07*, and EcN07** produced 133, 140, and 136 mg/L of α‐aminoadipate, respectively, Figure 5d).

### 3.5 Weakening the synthesis of acetic acid and realizing combination optimization

**Figure 6:**
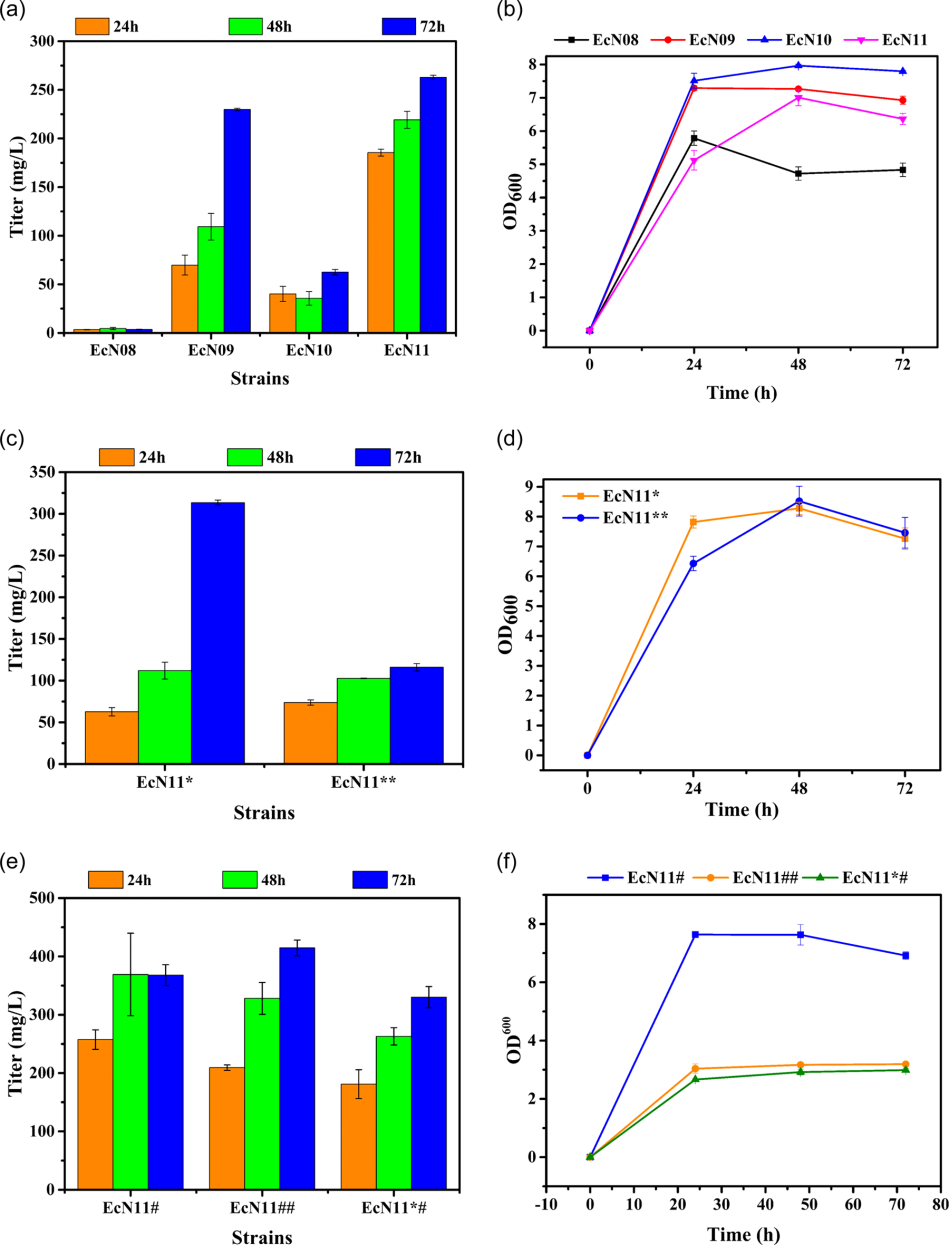
Inhibition of acetic acid synthesis and the combination optimization for improving α‐aminoadipate synthesis. (a), (c), and (e) The titers of α‐aminoadipate in the different strains. (b), (d), and (f) The biomass in the different strains.

**Figure B4:**
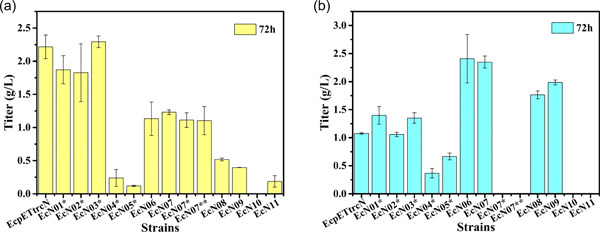
Acetic acid accumulation and glucose residuals in the different strains (a) The titers of acetic acid. (b) The titers of glucose.

**Figure B5:**
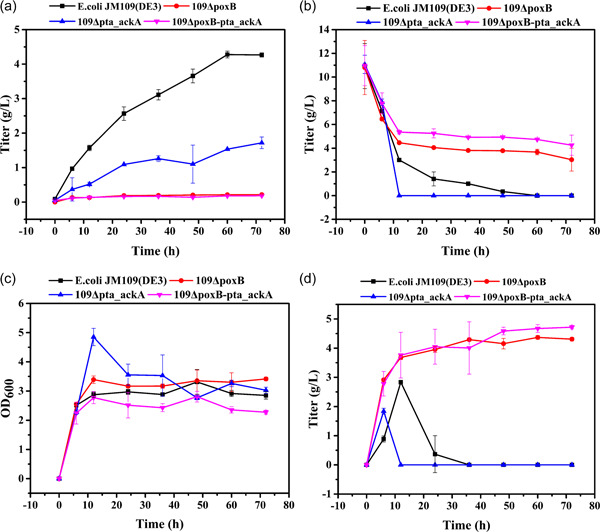
Acetic acid and pyruvate accumulation, glucose consumption, and the biomass changes of *E. coli* JM109(DE3) and its mutants in the M9 medium. (a) Acetic acid accumulation. (b) Glucose consumption. (c) Cell growth. (d) Pyruvate accumulation.

**Figure B6:**
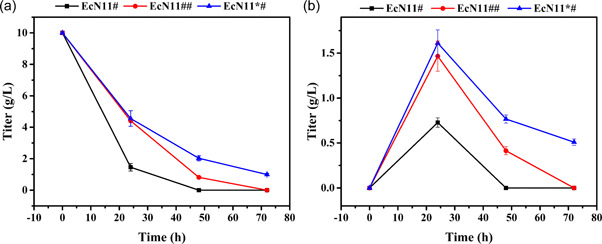
Glucose and pyruvate concentrations in strains EcN11#, EcN11##, and EcN11*#. (a) The titers of glucose (b) The titers of pyruvate.

**Figure B7:**
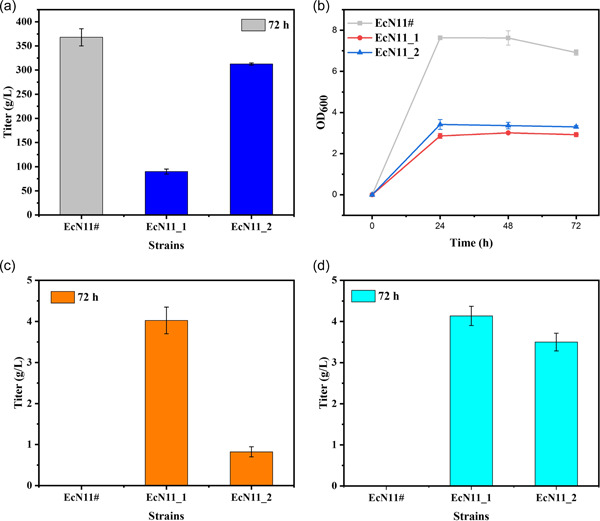
Metabolic flux analysis by experiments. (a) The titers of α‐aminoadipate in the different strains. (b) The biomass in the different strains. (c) The titers of pyruvate. (d) The titers of glucose.

Studies have shown that a large portion of the carbon source flows to the acetic acid synthesis pathway when *E. coli* uses glucose as the carbon source to express heterologous proteins (Bernal et al., 2016). The synthesis of acetic acid is a metabolic overflow phenomenon (Enjalbert et al., 2017). Under aerobic conditions, there are two primary ways to synthesize acetic acid. One is from pyruvate catalyzed by pyruvate oxidase (encoded by *poxB*). The other is from acetyl‐CoA catalyzed by phosphate acetyltransferase (encoded by *pta*) and acetate kinase (encoded by *ackA*) (Schütze et al., 2020). The fermentation results of the above 10 strains showed that acetic acid was the primary by‐product in the production of α‐aminoadipate by *E. coli*, resulting in many ineffective carbon fluxes (Appendix B, Figure B4). In addition, studies have shown that the knockout of acetic acid synthesis pathways can promote the synthesis of some compounds such as citramalate (Parimi et al., 2017). The FBA results also showed that the flux of α‐aminoadipate was negatively correlated with that of acetic acid. We first investigated the production of acetic acid, glucose consumption, and biomass changes of *E. coli* JM109(DE3) and its mutants in the M9 medium. The results showed that the knockout of *poxB* significantly inhibited acetic acid production, which is the key gene in acetic acid synthesis. However, the knockout of *poxB* affected glucose utilization, resulting in pyruvate accumulation (Figure B5). Subsequently, the *poxB* knockout strains were combined with the above regulatory strategies to promote the synthesis of α‐aminoadipate and obtain the strains EcN08‐EcN11. The strain EcN11 obtained the highest titer of α‐aminoadipate by fermentation in the shake flasks, up to 263 mg/L (Figure 6a), and this was 2.2 times higher than that of the strain EcpETtrcN. Hence, the knockout of the *poxB* gene and enhancement of pyruvate carboxylase effectively promoted glucose utilization and pyruvate conversion, reducing acetic acid flux (Figure B4). To reduce the number of plasmids and facilitate subsequent optimization, the *pycP458S* gene was integrated into the plasmid pET‐trc‐*lysDH‐pse* or pACYC‐tac‐*lysC311‐dapA_cg*, and the strains EcN11* and EcN11** were obtained. The fermentation results showed that the strain EcN11* produced a higher titer of α‐aminoadipate, up to 313.5 mg/L (Figure 6c). However, the titer of α‐aminoadipate produced by the EcN11** strain was lower than that produced by the strain EcN11, and glucose utilization and pyruvate conversion were blocked. At 72 h of fermentation, the glucose residue was 0.68 g/L, and pyruvate accumulated to 0.94 g/L. This may have been due to the low‐level expression of pyruvate carboxylase (Table A3). Finally, the titer of α‐aminoadipate was further improved by combination optimization. The strains EcN11#, EcN11#*, and Ec11*# are presented in (Table A3). The results demonstrated that the synthesis of α‐aminoadipate can be strengthened by combination optimization. The strains EcN11#, EcN11##, and Ec11*# produced 368, 415, and 330 mg/L of α‐aminoadipate, respectively (Figure 6e). Among them, the titers of α‐aminoadipate produced by the strains EcN11#, EcN11##, and EcN11*# strains were 17%, 32%, and 5% higher than that produced by the EcN11* strain, respectively. The carbon metabolisms of the EcN11## and EcN11*# strains were weakened due to the knockout of the *pgi* gene, and this negatively affected the growth and glucose absorption (Figures 6f and B6). To prove that the main flux of oxaloacetate in strain EcN11# came from the carbon fixation pathway catalyzed by pyruvate carboxylase, the main consumption pathway of pyruvate was blocked. In *E. coli*, pyruvate was mainly consumed in two pathways: (1) the conversion of pyruvate to acetic acid catalyzed by the pyruvate oxidase (encoded by *poxB*); (2) the conversion of pyruvate to acetyl‐CoA catalyzed by the pyruvate dehydrogenase complex (its rate‐limiting component was coded by *aceE*). Therefore, the flux of oxaloacetate was proved by the experiment of aceE knockout. We compared the growth and main metabolites of different strains. The results showed that the simultaneous knockout of *poxB* and *aceE* without the introduction of pyruvate carboxylase resulted in serious obstruction of glucose utilization, growth restriction, and serious accumulation of pyruvate, and the titer of α‐aminoadipate decreased seriously (Figure B7). This showed that pyruvate was mainly converted to acetyl‐CoA by the pyruvate dehydrogenase complex, and then entered the TCA cycle to produce oxaloacetate. To further explore the metabolic flux, strain EcN11_2 was obtained. The results showed the simultaneous knockout of *poxB* and *aceE* with the introduction of pyruvate carboxylase could reduce pyruvate accumulation and restore the titer of α‐aminoadipate. Although glucose utilization and strain growth were a little improved, they were still not as good as strain EcN11#. Thus, this proved that the most of metabolic flux of oxaloacetate in strain EcN11# was from pyruvate and only a small part of that was from the TCA cycle (Figure B7).

**Figure B8:**
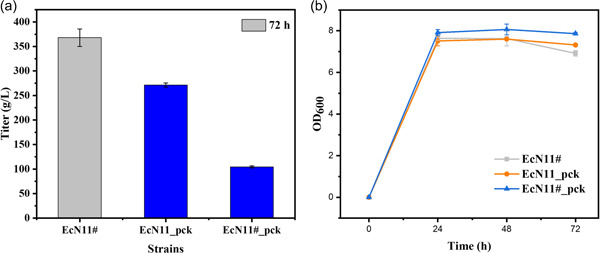
The effect of the removal of feedback inhibition at the anaplerotic pathway on the synthesis of α‐aminoadipate. (a) The titers of α‐aminoadipate in the different strains. (b) The biomass in the different strains.

In addition, we also explored the effect of the removal of feedback inhibition at the anaplerotic pathway on the synthesis of α‐aminoadipate through experiments. In *E. coli*, the conversion of oxaloacetate to PEP catalyzed by phosphoenolpyruvate carboxykinase (encoded by *pck*) could inhibit the replenishment of oxaloacetate, and excess oxaloacetate was introduced into gluconeogenesis through this reaction. We knocked out *pck* to the effect of the removal of feedback inhibition at the anaplerotic pathway on the synthesis of α‐aminoadipate. The results showed that the knockout of *pck* could promote the synthesis of α‐aminoadipate, but it was not as good as strengthening the replenishment pathway of oxaloacetate (The strain EcN11_pck, Figure B3). And knockout of *pck* and enhancement of oxaloacetate replenishment pathway resulted in the limited synthesis of α‐aminoadipate (The strain Ec11#_pck, Figure B3). This may be due to the imbalance of upstream and downstream metabolic flux and excessive OAA did not effectively enter the synthesis pathway of α‐aminoadipate but entered the TCA cycle (Figure B8).

### 3.6 Fed‐batch production of α‐aminoadipate

**Figure 7:**
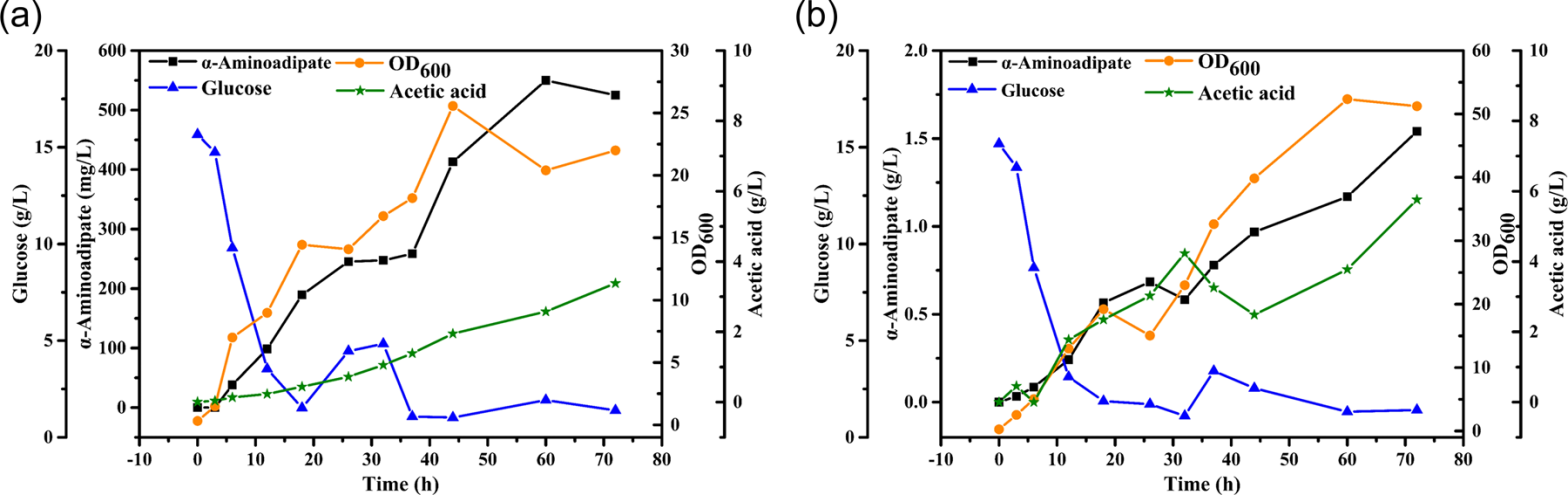
Fed‐batch production of α‐aminoadipate in 2 L bioreactors using strain EcN11#. DO was set at 10% (a) and 20% (b).

**Figure B9:**
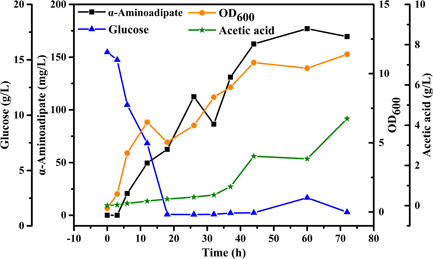
Fed‐batch production of α‐aminoadipate in 2 L bioreactors using strain EcN11##. DO was set at 10%.

To verify the scale‐up potential of α‐aminoadipate production, fed‐batch experiments were carried out in 2 L bioreactors using strains EcN11# and EcN11##. Initially, strain EcN11## was used for fed‐batch experiments. However, the results showed that strain EcN11## was not suitable for the scale‐up production of α‐aminoadipate (Figure B9), which is because its glycolytic pathway is blocked, resulting in the obstruction of glucose utilization and growth restriction. So it is difficult to realize high‐density fermentation. After that, we tested the ability of strain EcN11# to produce α‐aminoadipate. We investigated the effect of dissolved oxygen (DO) on α‐aminoadipate production. The results showed that high DO is not only beneficial for cell growth but also α‐aminoadipate accumulation. When DO was set at 10%, the cell density (OD_600_) of strain EcN11# only reached 20 and the final titer of α‐aminoadipate was only 525 mg/L at 72 h (Figure 7a). Furthermore, at 20% set DO, the cell density (OD_600_) reached 50 and 1.54 g/L of α‐aminoadipate was produced at 72 h (Figure 7b).

## 4 CONCLUSION

In this study, *E. coli* was successfully engineered to α‐aminoadipate from glucose. Two key enzymes, lysine dehydrogenase and aminoadipate semialdehyde dehydrogenase, from *Bacillus thermoamylovorans 1A1* and *Pseudomonas fulva 12‐X* were examined for the construction of an α‐aminoadipate biosynthesis pathway. The metabolic network was optimized by enhancing the synthesis of precursor lysine, introducing the CO_2_ fixation process, regulating intracellular cofactor metabolism, and inhibiting the synthesis of by‐product acetic acid. By using multi‐strategy metabolic engineering, the recombinant strains EcN11# and EcN11## improved α‐aminoadipate production from glucose. Shake flask fermentation using the strains EcN11# and EcN11## produced 368 and 415 mg/L of α‐aminoadipate. Finally, fed‐batch experiments produced 1.54 g/L of α‐aminoadipate by the strain EcN11#. This study is the first to achieve the green synthesis of α‐aminoadipate in microorganisms. To improve the yield of α‐aminoadipate from glucose, further metabolic engineering modifications would be required. At present, some studies have shown that improving transmembrane transport could strengthen mass transfer level and metabolic efficiency, and improve the production efficiency of cell factories (X. Chen et al., 2017; Fukui et al., 2019). So far, α‐aminoadipate transporter has not been reported. According to the structural similarity, a glutamate transporter may be used as α‐aminoadipate transporter. It has been reported that MscCG is a mechanosensitive channel of *Corynebacterium glutamicum* and acts as a glutamate transporter (Hashimoto et al., 2010; Nakayama et al., 2016, 2018; Yao et al., 2009). In addition, MscCG2 is a novel glutamate transporter found in *Corynebacterium glutamicum* which has a low amino acid sequence identity (23%) to MscCG (Wang et al., 2018). It was also demonstrated that constitutive glutamate effusion was triggered by its mutation of MscCG2 (A151V). We speculate that glutamate transporter is the possible transporter of α‐aminoadipate. Therefore, strengthening the expression of α‐aminoadipate transporter to improve its efflux could have a positive impact on improving the titer of α‐aminoadipate. Besides, some other metabolic engineering modifications could promote the synthesis of α‐aminoadipate, including stable expression of key genes through genomic integration, regulation of the tricarboxylic (TCA) cycle, identification of unknown and potential key metabolic nodes via omics analysis such as transcriptomics or metabolomics, etc.

## AUTHOR CONTRIBUTIONS

**Yang Zhang**: data curation (equal), project administration (equal), writing original draft (equal), writing review & editing (equal). **Meng Liu**: investigation (equal), methodology (equal). **Bingqi Cai**: software (equal). **Keqin He**: formal analysis (equal), visualization (equal). **Meng Wang**: funding acquisition (equal), resources (equal). **Biqiang Chen**: supervision (equal). **Tianwei Tan**: project administration (equal), supervision (equal).

## CONFLICT OF INTEREST

None declared.

## ETHICS STATEMENT

None required.

## Data Availability

All data are provided in full in this paper.

## APPENDIX A

(Tables A1, A2, A3)

**Table A1:** Strains and plasmids used in this study

Strain and plasmid	Relevant characteristic	Reference or source
Strain		
*E. coli* Trans 10	F‐mcrAΔ(mrr‐hsdRMS‐mcrBC)φ80 lacZΔM15ΔlacX74 recA1 araΔ139Δ(ara‐leu)7697 galUgalKrpsL(StrR) endA1 nupG	Purchased from TransGen Biotech
*E. coli* JM109(DE3)	endA1, recA1, gyrA96, thi, hsdR17 (rk‐, mk+), relA1, supE44, λ‐, Δ(lac‐proAB), [F', traD36, proAB, lacIqZΔM15], lDE3	Laboratory collection
109Δ*poxB*	Knockout of *poxB* gene in *E. coli* JM109(DE3)	Laboratory collection
109Δ*pta_ackA*	Knockout of *poxB* gene in *E. coli* JM109(DE3)	Laboratory collection
109Δ*poxB‐pta_ackA*	Knockout of *poxB, pta* and *ackA genes* in *E. coli* JM109(DE3)	Laboratory collection
109Δ*pgi*	Knockout of *pgi gene* in *E. coli* JM109(DE3)	Laboratory collection
109Δ*poxB*Δ*pgi*	Knockout of *poxB* and *pgi genes* in *E. coli* JM109(DE3)	Laboratory collection
*Corynebacterium glutamicum* ATCC 13032	Wild type	Laboratory collection
EcpETT7N	*E. coli* JM109(DE3) with pET‐T7‐*lysDH‐pse*	This study
EcpETtacN	*E. coli* JM109(DE3) with pET‐tac‐*lysDH‐pse*	This study
EcpETtrcN	*E. coli* JM109(DE3) with pET‐trc‐*lysDH‐pse*	This study
EcpETlacUV5N	*E. coli* JM109(DE3) with pET‐lacUV5‐*lysDH‐pse*	This study
EcpETlacO1N	*E. coli* JM109(DE3) with pET‐lacO1‐*lysDH‐pse*	This study
EcN01*	*E. coli* JM109(DE3) with pET‐trc‐*lysDH‐pse* and pACYC‐tac‐*lysC*_Ec‐*dapA*_Ec	This study
EcN02*	*E. coli* JM109(DE3) with pET‐trc‐*lysDH‐pse* and pACYC‐tac‐*lysCT311I* ‐*dapA*_cg	This study
EcN03*	*E. coli* JM109(DE3) with pET‐trc‐*lysDH‐pse* and pACYC‐PJ23100‐*ddh*_cg	This study
EcN04*	*E. coli* JM109(DE3) with pET‐trc‐*lysDH‐pse* and pRSF‐tac‐*pyc*	This study
EcN05*	*E. coli* JM109(DE3) with pET‐trc‐*lysDH‐pse* and pRSF‐tac‐*pycP458S*	This study
EcN05**	*E. coli* JM109(DE3) with pET‐trc‐*lysDH*‐*pse* and pRSF‐tac‐*ppc*_Ec	This study
EcN05*#	*E. coli* JM109(DE3) with pET‐trc‐*lysDH*‐*pse* and pRSF‐tac‐*ppc*_cg	This study
EcN05##	*E. coli* JM109(DE3) with pET‐trc‐*lysDH*‐*pse* and pRSF‐tac‐*pycP458S*‐*ppc*_cg	This study
EcN06	*E. coli* JM109(DE3) with pET‐trc‐*lysDH‐pse* and pCDF‐T7‐ED	This study
EcN07	*E. coli* JM109(DE3) with pET‐trc‐*lysDH‐pse* and pCDF‐T7‐*fdh1*‐T7‐*pntAB*	This study
EcN07*	109Δ*pgi* with pET‐trc‐*lysDH‐pse*	This study
EcN07**	109Δ*pgi* with pET‐trc‐*lysDH‐pse* and pCDF‐T7‐*fdh1*‐T7‐*pntAB*	This study
EcN08	*E. coli* JM109(DE3) with pET‐trc‐*lysDH‐pse* and pACYC‐tac‐*lysCT311I* ‐*dapA*_cg and pRSF‐tac‐*pycP458S*	This study
EcN09	109Δ*poxB* with pET‐trc‐*lysDH‐pse* and pACYC‐tac‐*lysCT311I* ‐*dapA*_cg	This study
EcN10	109Δ*poxB* with pET‐trc‐*lysDH‐pse* and pRSF‐tac‐*pycP458S*	This study
EcN11	109Δ*poxB* with pET‐trc‐*lysDH‐pse* and pACYC‐tac‐*lysCT311I* ‐*dapA*_cg and pRSF‐tac‐*pycP458S*	This study
EcN11*	109Δ*poxB* with pET‐trc‐*lysDH‐pse*‐tac‐*pycP458S* and pACYC‐tac‐*lysCT311I*‐*dapA*_cg	This study
EcN11**	109Δ*poxB* with pET‐trc‐*lysDH‐pse* and pACYC‐tac‐*lysCT311I* ‐*dapA*_cg‐tac‐*pycP458S*	This study
EcN11#	109Δ*poxB* with pET‐trc‐*lysDH‐pse*‐tac‐*pycP458S* and pACYC‐tac‐*lysCT311I*‐*dapA*_cg and pCDF‐T7‐*fdh1*‐T7‐*pntAB*	This study
EcN11##	109Δ*poxB*Δ*pgi* with pET‐trc‐*lysDH‐pse*‐tac‐*pycP458S* and pACYC‐tac‐*lysCT311I*‐*dapA*_cg	This study
EcN11*#	109Δ*poxB*Δ*pgi* with pET‐trc‐*lysDH‐pse*‐tac‐*pycP458S* and pACYC‐tac‐*lysCT311I* ‐*dapA*_cg and pCDF‐T7‐*fdh1*‐T7‐*pntAB*	This study
EcN11_1	109*ΔpoxBΔaceE* with pET‐trc‐*lysDH*‐*pse* and pACYC‐tac‐*lysCT311I*‐*dapA*_cg and pCDF‐T7‐*fdh1*‐T7‐*pntAB*	This study
EcN11_2	109*ΔpoxBΔaceE* with pET‐trc‐*lysDH*‐*pse‐tac‐pycP458S* and *pACYC‐tac‐lysCT311I*‐*dapA*_cg and pCDF‐T7‐*fdh1*‐T7‐*pntAB*	This study
EcN11_pck	109*ΔpoxBΔpck* with pET‐trc‐*lysDH*‐*pse* and pACYC‐tac‐*lysCT311I*‐*dapA*_cg and pCDF‐T7‐*fdh1*‐T7‐*pntAB*	This study
EcN11#_pck	109*ΔpoxBΔpck* with pET‐trc‐*lysDH*‐*pse‐tac‐pycP458S* and *pACYC‐tac‐lysCT311I*‐*dapA*_cg and pCDF‐T7‐*fdh1*‐T7‐*pntAB*	This study
Plasmid		
pUC57	*E. coli* cloning vector, pMB1 ori, Amp^R^	BGI collection
pUC57‐*lysDH*	pUC57 derivative; codon‐optimized *lysDH* gene from *Bacillus thermoamylovorans*, Amp^R^	This study
pUC57‐*pse*	pUC57 derivative; codon‐optimized *Psefu_1272* gene from *Pseudomonas fulva 12‐X*, Amp^R^	This study
pETDuet‐1	*E. coli* expression vector, T7 promoter, ColE1 ori, Amp^R^	Laboratory collection
pET‐tac‐T7‐T7ter	pETDuet‐1 derivative; tac and T7 promoters, ColE1 ori, Amp^R^	Laboratory collection
pACYCDuet‐1	*E. coli* expression vector, T7 promoter, P15A ori, Cm^R^	Laboratory collection
pACYC‐tac‐T7‐T7ter	pACYCDuet‐1 derivative; tac and T7 promoters, P15A ori, Cm^R^	Laboratory collection
pCDFDuet‐1	*E. coli* expression vector, T7 promoter, CloDF13 ori, Sm^R^	Laboratory collection
pRSFDuet‐1	*E. coli* expression vector, T7 promoter, RSF ori, Km^R^	Laboratory collection
pRSF‐tac‐T7‐T7ter	pRSFDuet‐1 derivative; tac and T7 promoters, RSF ori, Km^R^	Laboratory collection
pET‐T7‐*lysDH‐pse*	pETDuet‐1 derivative; T7 promoter, codon‐optimized *lysDH* gene from *Bacillus thermoamylovorans* and codon‐optimized *Psefu_1272* gene from *Pseudomonas fulva 12‐X*, ColE1 ori, Amp^R^	This study
pET‐tac‐*lysDH‐pse*	pET‐T7‐*lysDH‐pse* derivative; tac promoters, codon‐optimized *lysDH* gene from *Bacillus thermoamylovorans* and codon‐optimized *Psefu_1272* gene from *Pseudomonas fulva 12‐X*, ColE1 ori, Amp^R^	This study
pET‐trc‐*lysDH‐pse*	pET‐T7‐*lysDH‐pse* derivative; trc promoter, codon‐optimized *lysDH* gene from *Bacillus thermoamylovorans* and codon‐optimized *Psefu_1272* gene from *Pseudomonas fulva 12‐X*, ColE1 ori, Amp^R^	This study
pET‐lacUV5‐*lysDH‐pse*	pET‐T7‐*lysDH‐pse* derivative; lacUV5 promoter, codon‐optimized *lysDH* gene from *Bacillus thermoamylovorans* and codon‐optimized *Psefu_1272* gene from *Pseudomonas fulva 12‐X*, ColE1 ori, Amp^R^	This study
pET‐lacO1‐*lysDH‐pse*	pET‐T7‐*lysDH‐pse* derivative; lacO1 promoter, codon‐optimized *lysDH* gene from *Bacillus thermoamylovorans* and codon‐optimized *Psefu_1272* gene from *Pseudomonas fulva 12‐X*, ColE1 ori, Amp^R^	This study
pEASY‐Blunt Simple Cloning Vector	A cloning vector of *E. coli*, Kana^R^ or Amp^R^, pUC ori	Purchased from TransGen Biotech
pEASYblunt‐*lysC*	pEASY‐Blunt simple cloning vector derivative, Kana^R^ or Amp^R^, harboring aspartate kinase encoding gene *lysC*	This study
pEASYblunt‐ *lysCT311I*	pEASY‐Blunt simple cloning vector derivative, Kana^R^ or Amp^R^, harboring the mutant of aspartate kinase encoding gene *lysCT311I*	This study
pACYC‐tac‐*lysC*_Ec‐*dapA*_Ec	pET‐tac‐T7‐T7ter derivative; tac promoter, *lysC* and *dapA* genes from *E. coli* JM109(DE3), P15A ori, Cm^R^	This study
pACYC‐tac‐*lysCT311I* ‐*dapA*_cg	pACYC‐tac‐T7‐T7ter derivative; tac promoter, *lysC311I* and *dapA* genes from *C. glutamicum* ATCC 13032, P15A ori, Cm^R^	This study
pACYC‐PJ23100‐*ddh*_cg	pACYCDuet‐1 derivative; PJ23100 promoter, *ddh* gene from *Corynebacterium glutamicum* ATCC 13032, P15A ori, Cm^R^	This study
pRSF‐tac‐*pyc*	pRSF‐tac‐T7‐T7ter derivative; tac promoter, *pyc* gene from *C. glutamicum* ATCC 13032, RSF ori, Km^R^	This study
pRSF‐tac‐*pycP458S*	pRSF‐tac‐T7‐T7ter derivative; tac promoter, *pyc* gene mutant, RSF ori, Km^R^	This study
pRSF‐tac‐*ppc*_Ec	pRSF‐tac‐T7‐T7ter derivative; tac promoter, *ppc* gene from *E. coli JM109(DE3)*, RSF ori, KmR	This study
pRSF‐tac‐*ppc*_cg	pRSF‐tac‐T7‐T7ter derivative; tac promoter, *ppc* gene from *Corynebacterium glutamicum* ATCC 13032, RSF ori, KmR	This study
pRSF‐tac‐*pycP458S*‐*ppc*_cg	pRSF‐tac‐T7‐T7ter derivative; tac promoter, *pyc* gene mutant, *ppc* gene from *Corynebacterium glutamicum* ATCC 13032, RSF ori, KmR	This study
pCDF‐T7‐ED	pCDFDuet‐1 derivative; T7 promoter, *zwf*、*pgl*、*edd* and *eda* genes from *Zymomonas mobilis subsp. Mobilis* CICC 41465, CloDF13 ori, Sm^R^	Laboratory collection
pCDF‐T7‐*fdh1*‐T7‐*pntAB*	pCDFDuet‐1 derivative; T7 promoter, *fdh1* gene from *Saccharomyces cerevisiae s288c*, *pntAB* gene from *E. coli* JM109(DE3), CloDF13 ori, Sm^R^	Laboratory collection
pET‐trc‐*lysDH**‐**pse*‐tac‐*pycP458S*	pET‐trc‐*lysDH‐pse* derivative; trc promoter, codon‐optimized *lysDH* gene from *Bacillus thermoamylovorans* and codon‐optimized *Psefu_1272* gene from *Pseudomonas fulva 12‐X*; tac promoter, *pyc* gene mutant, ColE1 ori, Amp^R^	This study
pACYC‐tac‐*lysC311‐dapA_cg*‐ tac‐*pycP458S*	pACYC‐tac‐*lysC311‐dapA_cg* derivative; tac promoter, *lysC311I* and *dapA* genes from *Corynebacterium glutamicum* ATCC 13032; tac promoter, *pyc* gene mutant, P15A ori, Cm^R^;	This study

**Table A2:** List of primers used in this study

Primers	Sequence (5’–3’)	Descriptions
pET‐*lysDH*‐Gibson‐F	ACTTTAAGAAGGAGATATACCATGAAAATTGGCGTGCTGG	Amplification of gene *lysDH*
pET‐*lysDH*‐Gibson‐R	TATATCTCCTTTAGCTCAGATTTTCGCTAATCACC	
pET‐*pse*‐Gibson‐F	TCTGAGCTAAAGGAGATATACCATGGTGAATAGCCTGCTGG	Amplification of gene *pse*
pET‐*pse*‐Gibson‐R	TTTACCAGACTCGAGGGTACCTTAATCAAAAACAATGCCCTGCG	
lacI‐ApaI‐F	TTAAGGGCCCGCTAACAGCGCGATTTGCTG	Amplification of gene lacI
tac‐XbaI‐R	CTAGTCTAGAGGGGAATTGTTATCCGCTCACAATTCCACACATTATACGAGCCGATGATTAATTGTCAATTTCGCGGGATCGAGATC	Amplification of tac promoter
trc‐XbaI‐R	CTAGTCTAGAGGGGAATTGTTATCCGCTCACAATTCCCATTATACGAGCCGGATGATTAATTGTCAAATTTCGCGGGATCGAGATC	Amplification of trc promoter
lacUV5‐XbaI‐R	CTAGTCTAGAGGGGAATTGTTATCCGCTCACAATTCCCATTATACGAGCCGGAAGCATAAAGTGTAAAATTTCGCGGGATCGAGATC	Amplification of lacUV5 promoter
lacO1‐XbaI‐R	CTAGTCTAGAGGGGTCAGTGCGTCCTGCTGATGTGCTCAGTATCTTGTTATCCGCTCACAATGTCAATTGTTATCCGCTCACAATTATTTCGCGGGATCGAGATC	Amplification of lacO1 promoter
*lysC*‐F	GTGGCCCTGGTCGTACAG	Amplification of gene *lysC* from *Corynebacterium glutamicum* ATCC 13032
*lysC‐R*	TTAGCGTCCGGTGCCTGC	
*lysCT311I*‐F	CATCATCTTCACCTGCCCT	Amplification of gene *lysCT311I*
*lysCT311I*‐R	TCGGTGGTGCCGTCTTCTA	
pACYC‐*lysC*_Ec‐HindIII‐F	CCCAAGCTTATGTCTGAAATTGTTGTCTCCAAATTTGG	Amplification of gene *lysC* from *E. coli* JM109(DE3)
pACYC‐*lysC*_Ec‐PacI‐R	CCTTAATTAATTACTCAAACAAATTACTATGCAGTTTTTGC	
pACYC‐RBS‐*dapA*_Ec‐PacI‐F	CCTTAATTAAAGGAGATATACCATGTTCACGGGAAGTATTGTCG	Amplification of gene *dapA* from *E. coli* JM109(DE3)
pACYC‐*dapA*_Ec‐AvrII‐R	GGGAAACCTAGGTTACAGCAAACCGGCATGC	
pACYC‐*lysCT311I* _cg‐SalI‐F	ACGCGTCGACATGGCCCTGGTCGTACAGA	Amplification of gene *lysCT311I*
pACYC‐*lysCT311I* _cg‐PacI‐R	CCTTAATTAATTAGCGTCCGGTGCCTGC	
pACYC‐RBS‐*dapA*_cg‐PacI‐F	CCTTAATTAAAGGAGATATACCATGAGCACAGGTTTAACAGCTAAG	Amplification of gene *dapA* from *Corynebacterium glutamicum* ATCC 13032
pACYC‐*dapA*_cg‐AvrII‐R	GGGAAACCTAGGTTATAGAACTCCAGCTTTTTTCATGTCTTC	
pRSF‐*pyc*‐HindII‐F	CCCAAGCTTATGTCGACTCACACATCTTCAACG	Amplification of gene *pyc* from *Corynebacterium glutamicum* ATCC 13032
pRSF‐*pyc*‐AvrrII‐R	GGGAAACCTAGGTTAGGAAACGACGACGATCAAGTCG	
*pycP458S*‐R	GTGATCGGCAATGAATCCGG	Amplification of gene *pycP458S*
*pycP458S*‐F	CCGGATTCATTGCCGATCACTCGCACCTCCTTCAGGCTC	
T7ter‐NsiI‐tac‐AvrII‐F	GGGAAACCTAGGCTAGCATAACCCCTTGGGGCCTCTAAACGGGTCTTGAGGGGTTTTTTGATGCATTTGACAATTAATCATCGGCTCG	Amplification of gene *pyc* or *pycP458S*

**Table A3:** Glucose, acetic acid, and pyruvate concentration at 72 h in strains EcN11* and EcN11*

Strains	The titer of glucose (g/L)	The titer of acetic acid (g/L)	The titer of pyruvate (g/L)
EcN11*	N.A.	N.A.	N.A.
EcN11**	0.68	N.A.	0.94

Abbreviation: N.A., not available.

## APPENDIX B

(Figures B1, B2, B3, B4, B5, B6, B7, B8, B9)
